# Immune responses against SARS-CoV-2 variants after heterologous and homologous ChAdOx1 nCoV-19/BNT162b2 vaccination

**DOI:** 10.1038/s41591-021-01449-9

**Published:** 2021-07-14

**Authors:** Joana Barros-Martins, Swantje I. Hammerschmidt, Anne Cossmann, Ivan Odak, Metodi V. Stankov, Gema Morillas Ramos, Alexandra Dopfer-Jablonka, Annika Heidemann, Christiane Ritter, Michaela Friedrichsen, Christian Schultze-Florey, Inga Ravens, Stefanie Willenzon, Anja Bubke, Jasmin Ristenpart, Anika Janssen, George Ssebyatika, Günter Bernhardt, Jan Münch, Markus Hoffmann, Stefan Pöhlmann, Thomas Krey, Berislav Bošnjak, Reinhold Förster, Georg M. N. Behrens

**Affiliations:** 1 Institute of Immunology, Hannover Medical School, Hannover, Germany; 2 Department for Rheumatology and Immunology, Hannover Medical School, Hannover, Germany; 3 German Center for Infection Research (DZIF), Partner Site Hannover-Braunschweig, Hannover, Germany; 4 Department of Hematology, Hemostasis, Oncology and Stem-Cell Transplantation, Hannover Medical School, Hannover, Germany; 5 Institute of Biochemistry, University of Lübeck, Lübeck, Germany; 6 Ulm University Medical Center, Institute of Molecular Virology, Ulm, Germany; 7 Infection Biology Unit, German Primate Center, Göttingen, Germany; 8 Faculty of Biology and Psychology, Georg-August-University Göttingen, Göttingen, Germany; 9 German Center for Infection Research (DZIF), Partner Site Hannover-Braunschweig and Partner Site Hamburg-Lübeck-Borstel-Riems, Hamburg, Germany; 10 Cluster of Excellence RESIST (EXC 2155), Hannover Medical School, Hannover, Germany; 11 CiiM, Centre for Individualised Infection Medicine, Hannover, Germany

**Keywords:** Vaccines, Medical research, T cells, B cells, SARS-CoV-2

## Abstract

Currently approved viral vector-based and mRNA-based vaccine approaches against coronavirus disease 2019 (COVID-19) consider only homologous prime-boost vaccination. After reports of thromboembolic events, several European governments recommended using AstraZeneca’s ChAdOx1-nCov-19 (ChAd) only in individuals older than 60 years, leaving millions of already ChAd-primed individuals with the decision to receive either a second shot of ChAd or a heterologous boost with mRNA-based vaccines. However, such combinations have not been tested so far. We used Hannover Medical School’s COVID-19 Contact Study cohort of healthcare professionals to monitor ChAd-primed immune responses before and 3 weeks after booster with ChAd (*n* = 32) or BioNTech/Pfizer’s BNT162b2 (*n* = 55). Although both vaccines boosted prime-induced immunity, BNT162b2 induced significantly higher frequencies of spike-specific CD4^+^ and CD8^+^ T cells and, in particular, high titers of neutralizing antibodies against the B.1.1.7, B.1.351 and P.1 variants of concern of severe acute respiratory syndrome coronavirus 2.

## Abstract (web summary)

In a study of healthcare professionals previously vaccinated with ChAdOx-1 nCoV-19, booster vaccination with BNT162b2 elicited more neutralizing antibodies with greater breadth, as well as higher frequencies of virus-specific T cells, than ChAdOx-1 nCoV-19.

## Main

The first approved vaccine against COVID-19 was the lipid nanoparticle-formulated mRNA vaccine BNT162b2 (Comirnaty, BNT), which was developed by BioNTech/Pfizer. BNT was proven safe and 95% effective in preventing COVID-19 (ref. ^1^). Similarly, ChAdOx1-nCov-19 (Vaxzevria, ChAd), a replication-deficient chimpanzee adenovirus-vectored vaccine developed by Oxford University in collaboration with AstraZeneca, had an acceptable safety profile, albeit with a somewhat lower efficacy of 70.4% against symptomatic COVID-19 (ref. ^2^). These data, together with the efficacy of other vaccines, including those from Moderna^3^ and Johnson & Johnson^4^, raised hope for expeditious ending of the severe acute respiratory syndrome coronavirus 2 (SARS-CoV-2) pandemic.

However, in the first half of March 2021, vaccinations with ChAd were halted after reports of moderate to severe thrombocytopenia and unusual thrombosis cases in vaccinees^5,7^. This new syndrome, termed vaccine-induced thrombotic thrombocytopenia^6^ or thrombosis with thrombocytopenia syndrome, appears to be induced by antibodies directed against platelet factor 4 that lead to platelet activation^8^. Despite concerns, the European Medicines Agency concluded that the benefits of ChAd vaccination outweigh the potential risks for an individual (https://www.ema.europa.eu/en/news/astrazenecas-covid-19-vaccine-benefits-risks-context; accessed 17 June 2021), and ChAd remains a valuable tool against COVID-19. However, many countries recommended to vaccinees, who received the first ChAd dose, to have an mRNA vaccine or to choose between ChAd-based or mRNA-based vaccines as a second (boost) dose. An initial report from the United Kingdom randomized Com-COV Study suggested more short-term reactogenicity of heterologous prime-boost schedules^9^.

Furthermore, mutations in the SARS-CoV-2 spike protein lead to rapidly expanding variants of concern (VoC), including B.1.1.7 (Alpha), B.1.351 (Beta), P.1 (Gamma, formerly named B.1.1.28.1) and B.1.617.2 (Delta) variants^10^, which raised concerns about containing the SARS-CoV-2 variants through vaccination. Antibodies induced by BNT and ChAd vaccines efficiently neutralize the B.1.1.7 variant, and the neutralization of P.1 and B.1.351 variants seems to be reduced^11–13^. Moreover, BNT vaccination has been shown to be approximately 13% and 28% less protective against development of symptomatic COVID-19 for variants B.1.1.7 and B.1.351, respectively^14^. Similarly, it has been reported that protection from symptomatic COVID-19 after ChAd vaccination is slightly reduced for the B.1.1.7 variant^15^, whereas no protection against mild to moderate COVID-19 caused by the B.1.351 variant was observed^16^. It remains to be determined whether heterologous prime-boost regimens can induce equal or even stronger immune responses against the novel viral variants compared to the homologous prime-boost regimens.

**Fig. 1:**
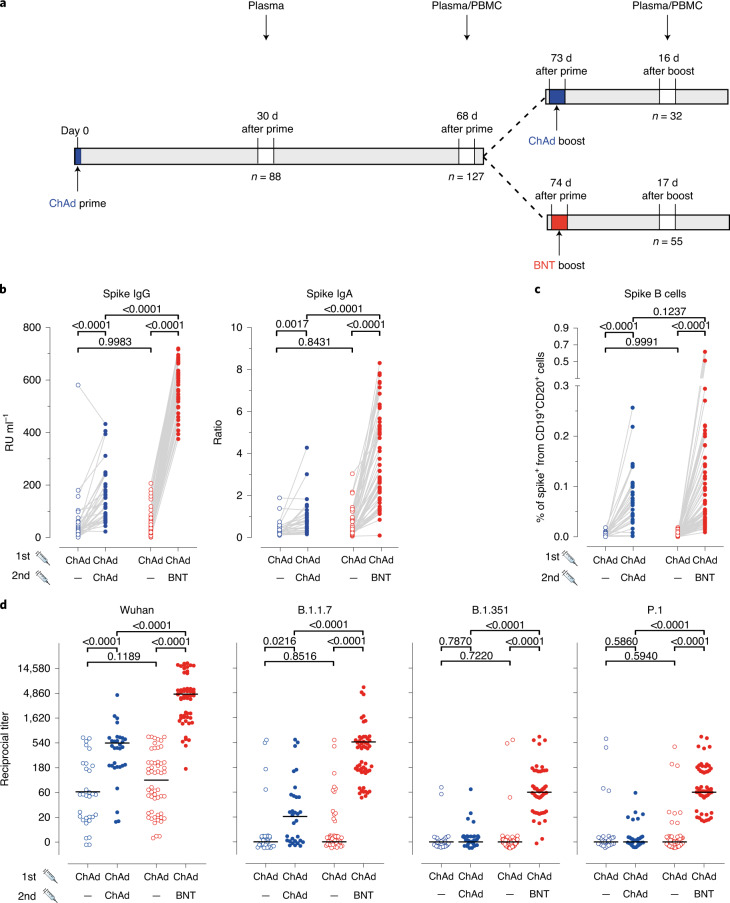
Stronger humoral immune responses against all SARS-CoV-2 variants after heterologous ChAd/BNT versus homologous ChAd/ChAd vaccination. **a**, Participant recruitment scheme. **b**, S-specific IgG and IgA levels in plasma after prime (open circles) and after boost (closed circles) from homologous ChAd/ChAd (blue symbols) and heterologous ChAd/BNT (red symbols) vaccinees. Data are from *n* = 32 biologically independent samples from the ChAd/ChAd group and *n* = 55 biologically independent samples from the ChAd/BNT group. **c**, Percentage of spike-specific from total B cells in the whole blood measured using flow cytometry. Data are from *n* = 32 biologically independent samples from the ChAd/ChAd group and *n* = 55 biologically independent samples from the ChAd/BNT group. **d**, Reciprocal titers of neutralizing antibodies against Wuhan, B.1.1.7 (Alpha), P.1 (B.1.1.28.1; Gamma) and B.1.351 (Beta) SARS-CoV-2-S variants measured using the sVNT. Data are from *n* = 31 biologically independent samples from the ChAd/ChAd group and *n* = 54 biologically independent samples from the ChAd/BNT group. For better visualization of identical titer values, data were randomly and proportionally adjusted closely around the precise titer results. Statistics: **b** and **c**. Paired *t*-test (within groups) or two-way ANOVA followed by Sidak’s multiple comparison test (between groups). **d**: chi-square test for trend. **b**–**d**: Dots represent individual vaccinees; lines represent group median. Source data

**Extended Data Fig. 1:**
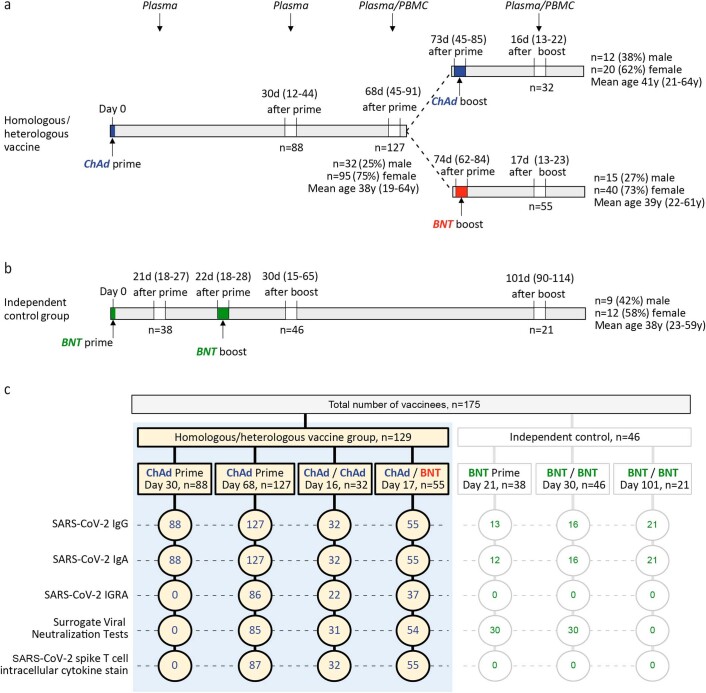
Participant recruitment schemes. **a**, Participant recruitment scheme including age and sex information. Days are given as mean (range). **b**, Participant recruitment scheme for vaccinees immunized twice with BNT vaccine. **c**, Scheme indicating number of samples analyzed with each assay in each group.

**Extended Data Fig. 2:**
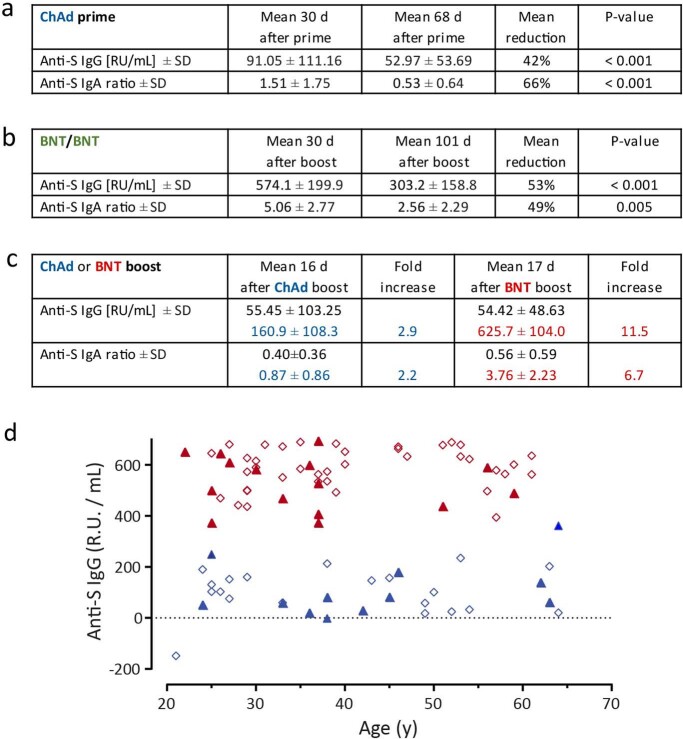
Mean anti-S IgG and IgA values in study groups. **a**, Time course of anti-S IgG and IgA decline over mean 38 days after ChAd prime and before full BNT vaccination (n = 86). Differences were assessed by paired t-test. **b**, Time course of anti-S IgG and IgA decline over mean 71 days in the independent control group after BNT/BNT full vaccination (n = 21). Differences were assessed by paired t-test. **c**, Fold increase of anti-S IgG and IgA after ChAd prime (top values) followed by either ChAd (n = 32) or BNT (n = 55) booster vaccination (blue or red values). **d**, Increase in anti-S IgG after ChAd prime followed by either ChAd (blue) or BNT (red) booster vaccination in relation to age and sex (male = triangles, female=rhombs). It should be noted that anti-S IgG differences for heterologous vaccination were always greater than for homologous vaccination. Source data

To analyze the efficacy of the heterologous prime-boost vaccination schedule, we used our COVID-19 Contact (CoCo) Study cohort of healthcare professionals (HCPs)^17,18^ and monitored responses to homologous and heterologous prime-boost COVID-19 vaccine treatment schedules (Methods). Hannover Medical School HCP vaccinees who received one dose of ChAd were offered a choice between the ChAd and BNT vaccines for a second dose. To determine immunogenicity of the homologous and heterologous immune regimens, we studied 129 ChAd-primed vaccinees without previous SARS-CoV-2 infection, of whom 32 chose homologous boosting and 55 chose heterologous boosting. For comparison, we included a group of 46 BNT/BNT vaccinated HCPs. The vaccination and blood collection schedule is depicted in Fig. 1a, with additional demographic information (age and sex) in Extended Data Fig. 1a–c. A retrospective analysis revealed that the mean anti-SARS-CoV-2 spike IgG (anti-S IgG) and IgA had declined by 42% and 66%, respectively, from mean titers 30 d after ChAd prime to shortly before boosting, which is similar to declines in BNT/BNT vaccinated individuals (Extended Data Fig. 2a,b). Notably, we found similar levels of anti-S IgG and IgA antibodies in the ChAd/ChAd and the ChAd/BNT groups before the booster, indicating that both groups responded equally well after priming with ChAd (Fig. 1b).

**Extended Data Fig. 3:**
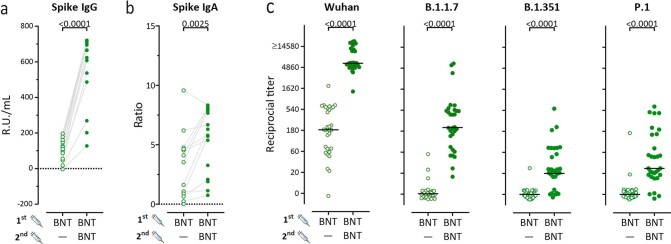
Humoral immune response against all SARS-CoV-2 variants following homologous BNT162b2 (BNT) / BNT162b2 (BNT) vaccination. **a**,**b**, Spike-specific IgG (**a**) and IgA (**b**) levels in plasma after prime (open circles) and 30 days (mean) after booster (closed circles) homologous BNT/BNT vaccination. Data are from n = 16 biologically independent sample pairs. **c**, Reciprocal titers of neutralizing antibodies against Wuhan-Spike, B.1.1.7-Spike (Alpha), P.1-Spike (B.1.1.28.1; Gamma), and B.1.351-Spike (Beta) SARS-CoV-2 variants measured using surrogate virus neutralization test (sVNT). Data are from n = 30 biologically independent sample pairs. For better visualization of identical titer values, data were randomly and proportionally adjusted closely around the precise titer results. Statistics: a-b. Paired t-test (within groups); c. Chi-square test for trend. **a**-**c**, Dots represent individual vaccines, lines represent group median. Source data

After the booster immunization, increased anti-spike (S) IgG and IgA responses were found in both groups. Heterologous ChAd/BNT vaccination led to a significant 11.5-fold increase for anti-S IgG (*P* < 0.0001) compared to a 2.9-fold increase after homologous ChAd vaccination (*P* < 0.0001) (Fig. 1b and Extended Data Fig. 2c). Differences in anti-S IgG were not significantly influenced by age or sex of participants (Extended Data Fig. 2d). We observed similar changes for anti-S IgA (Fig. 1b), indicating better humoral immune responses after heterologous prime-boost immunization. Anti-S IgG and IgA concentrations after ChAd/BNT vaccination were within the range of fully BNT/BNT vaccinated individuals (Extended Data Fig. 2b and Extended Data Fig. 3a,b).

**Extended Data Fig. 4:**
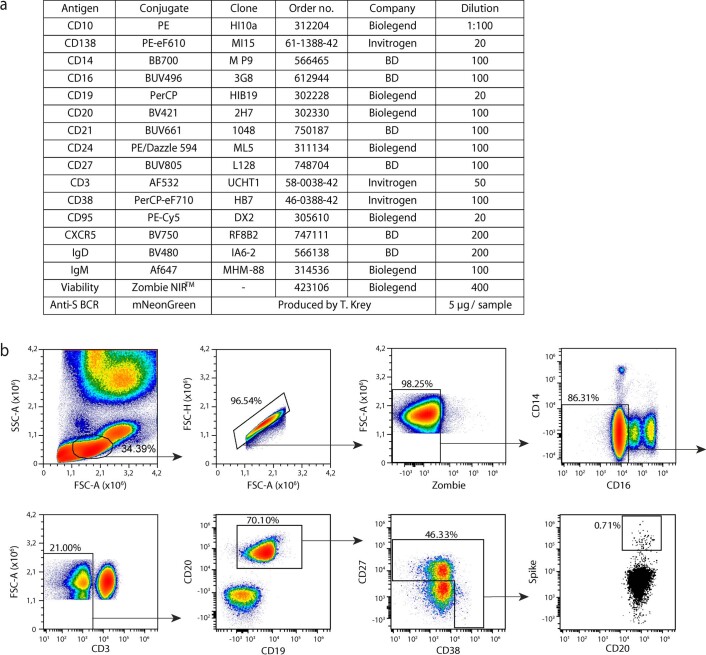
Antibody panel (a) and gating strategy (b) for SARS-CoV-2-S (Spike)-specific B cell populations in blood. Pseudocolor plots show representative data from a female donor 71 days after priming with ChAd and 20 days post boost with BNT.

We next determined the frequency and phenotype of B cells carrying membrane-bound immunoglobulins specific for the spike protein (Methods and Extended Data Fig. 4). Interestingly, in samples taken before booster vaccination, spike-specific memory B cells could be detected in only 53.1% (17/32) of vaccinees from the ChAd/ChAd group and in only 43.6% (24/55) of vaccinees from the ChAd/BNT group. Moreover, if present, spike-specific memory B cells represented only a rare (~0.003%) population of whole-blood B cells, with no significant difference between the ChAd/ChAd and the ChAd/BNT groups (Fig. 1c, open circles). In contrast, spike-specific memory B cells were significantly increased in all vaccinees from both the ChAd/ChAd and ChAd/BNT groups after booster vaccination (Fig. 1c, filled dots). In contrast to the anti-S antibody responses, heterologous ChAd/BNT and homologous ChAd/ChAd vaccinations led to expansion of spike-specific memory B cells to a similar extent. The increased frequencies of spike-specific memory B cells after booster immunization, combined with increased amounts of spike-specific antibodies, highlight the importance of the booster vaccination for full protection from SARS-CoV-2 infection.

**Extended Data Fig. 5:**
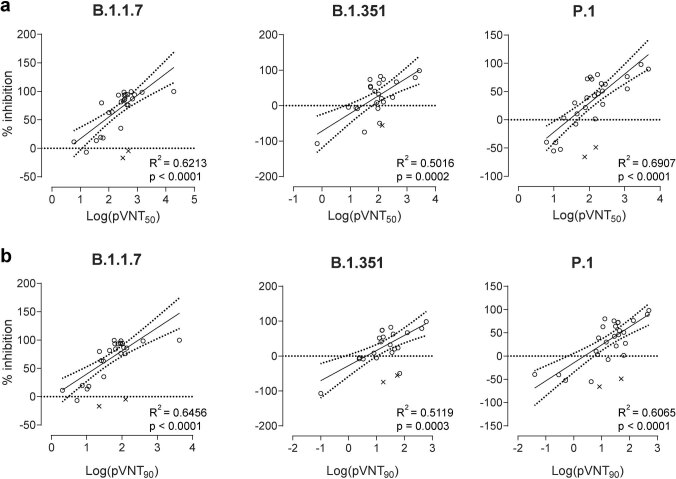
Antibody neutralization measurements against different SARS-CoV-2 variants are positively correlated between the virus neutralization tests (sVNT) and pseudotyped virus neutralization tests (pVNT). Correlation (solid line) and 95% confidence intervals (dotted lines) between sVNT_1:20_ and antibody titers resulting in 50% (**a**) or 90% (**b**) reduction of luciferase activity in pVNT, indicated as pVNT_50_ and pVNT_90_, respectively. Open circles, values from individual donors, outliers are marked with X and were defined as values with absolute residual value > 2 SD of all residual values in each group of samples. Correlation was calculated using single linear regression. Source data

To test for neutralizing activity of antibodies induced by infection or vaccination, we recently developed an enzyme-linked immunosorbent assay (ELISA)-based surrogate virus neutralization test (sVNT)^19^. We adapted the sVNT to include spike proteins of the B.1.1.7, P.1 and B.1.351 VoC (Methods). To validate these new assays, we applied sera from vaccinees who had been recently tested for their neutralizing capacity, applying the vesicular stomatitis virus (VSV)-based pseudotyped virus neutralization test (pVNT)^12^. Comparing results obtained using pVNT with those of the newly developed sVNTs, we observed a high degree of correlation between both assays, with *R*^2^ values ranging between 0.50 and 0.69 (Extended Data Fig. 5). These findings demonstrate that the sVNT is suited to quantitatively assess the neutralization capacity of vaccination-induced antibodies, not only against the Wuhan strain but also against the B.1.1.7, P.1 and B.1.351 variants of SARS-CoV-2.

**Extended Data Fig. 6:**
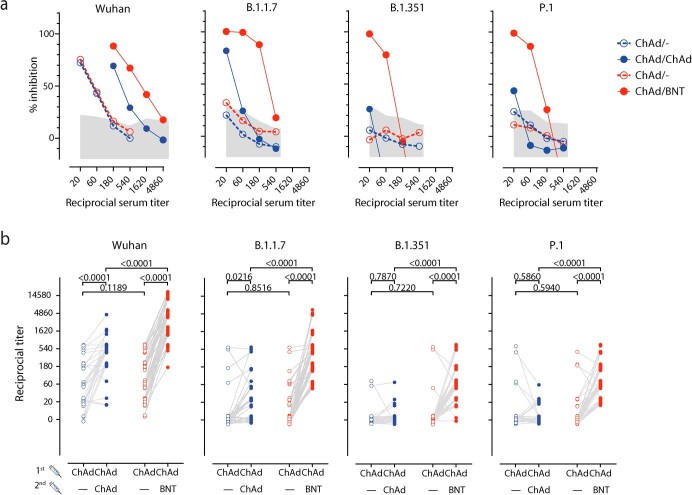
Neutralizing antibodies interfere with binding of SARS-CoV-2-S-RBD variants to human ACE2. **a**, Inhibition of interaction of indicated SARS-CoV-2-S-RBD variants with ACE2 by the addition of plasma of a representative vaccinee before (open circles) and after (closed circles) homologous ChAd/ChAd (blue symbols) and heterologous ChAd/BNT (red symbols) booster immunization. Assays were performed in duplicates and are shown as mean percentages of neutralization. Shaded areas represent mean +2 SD of values from pre-COVID-19 plasma. **b**, Reciprocal titers of neutralizing antibodies against the Wuhan-Spike, B.1.1.7-Spike (Alpha), P.1-Spike (B.1.1.28.1; Gamma), and B.1.351-Spike (Beta) SARS-CoV-2 variants in plasma of each vaccinee before and after the boost immunization. Statistics: Chi-square test for trend. Data are from n = 31 biologically independent sample pairs from the ChAd/ChAd group and n = 54 biologically independent sample pairs from the ChAd/BNT group. For better visualization of identical titer values, data were randomly and proportionally adjusted closely around the precise titer results. Source data

Applying sVNT assays, we found that 81 of 88 participants had neutralizing antibodies against the Wuhan strain in pre-boost plasma. In contrast, neutralizing antibodies against the B.1.1.7 (17/88), P.1 (12/88) and B.1.351 (5/88) variants were less frequent (Fig. 1d and Extended Data Fig. 6). At 2–3 weeks after the booster immunization, frequencies and titers of neutralizing antibodies against the Wuhan strain increased in the ChAd/ChAd and ChAd/BNT groups, with titers reaching higher values in the latter group (Fig. 1d and Extended Data Fig. 6). Differences between the ChAd and the BNT booster vaccination became even more evident when analyzing the neutralization capacity of antibodies induced against the VoC. In the ChAd/ChAd group, booster immunization increased neutralization of the B.1.1.7 variant in some individuals but showed no effect against the P.1 and B.1.351 variants (Fig. 1d and Extended Data Fig. 6). In contrast, booster immunization with BNT induced neutralizing antibodies at high frequencies against all analyzed VoC. In the ChAd/BNT group, all participants had neutralizing antibodies against the B.1.1.7 and P.1 variants, and all but two participants also had neutralizing antibodies against the B.1.351 variant (Fig. 1d and Extended Data Fig. 6). In the ChAd/BNT group, the post-boost neutralization capacity was highest against the Wuhan strain, followed by the B.1.1.7 variant and less efficient against the P.1 and B.1.351 variants (Fig. 1d and Extended Data Fig. 6). Altogether, these data indicate that the booster immunization led to an increase of neutralizing antibodies in both vaccination groups and that the heterologous BNT booster vaccination efficiently induced neutralizing antibodies against all tested VoC.

**Fig. 2:**
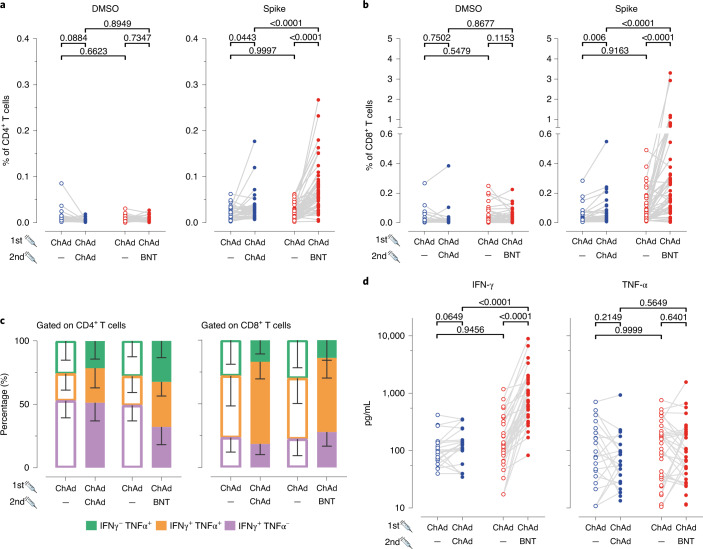
Heterologous ChAd/BNT vaccination induces stronger anti-SARS-CoV-2 spike T cell responses versus homologous ChAd/ChAd vaccination. **a**, **b**, Boost vaccination increased total percentage of cytokine-secreting CD4^+^ (**a**) and CD8^+^ (**b**) T cells. We calculated the total number of cytokine-secreting cells as the sum of IFN-γ^+^TNF-α^−^, IFN-γ^+^TNF-α^+^ and IFN-γ^−^TNF-α^+^ cells in the gates indicated in Extended Data Fig. 7. Data are from *n* = 32 biologically independent samples from the ChAd/ChAd group and *n* = 55 biologically independent samples from the ChAd/BNT group. **c**, Increased percentage of double cytokine-secreting CD4^+^ and CD8^+^ T cells after the second vaccine dose. Data are from *n* = 32 biologically independent samples from the ChAd/ChAd group and *n* = 55 biologically independent samples from the ChAd/BNT group. **d**, IFN-γ and TNF-α concentration in full blood supernatants after stimulation with SARS-CoV-2 S1 domain for 20–24 h measured in duplicate by LEGENDplex (BioLegend). Data are from *n* = 22 biologically independent samples from the ChAd/ChAd group and *n* = 37 biologically independent samples from the ChAd/BNT group. Statistics: **a**, **b** and **d**. Paired *t*-test (within groups) or two-way ANOVA followed by Sidak’s multiple comparison test (between groups). Dots represent individual vaccinees. **c**: Data are represented as mean − s.d. Source data

**Extended Data Fig. 7:**
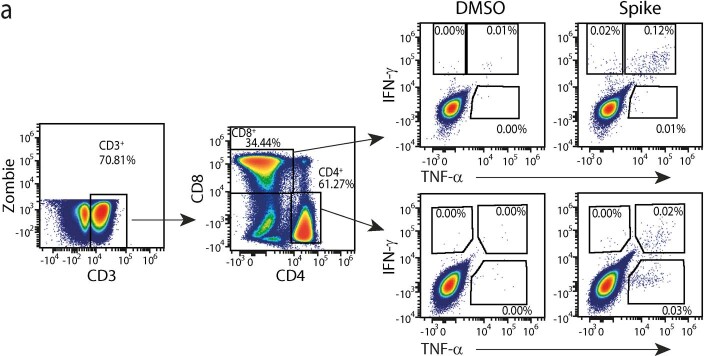
Gating strategy used for detection of cytokine producing CD4^+^ and CD8^+^ T cells after *ex vivo* re-stimulation with DMSO or the pool of Spike-specific peptides for 12–16 hr.

**Extended Data Fig. 8:**
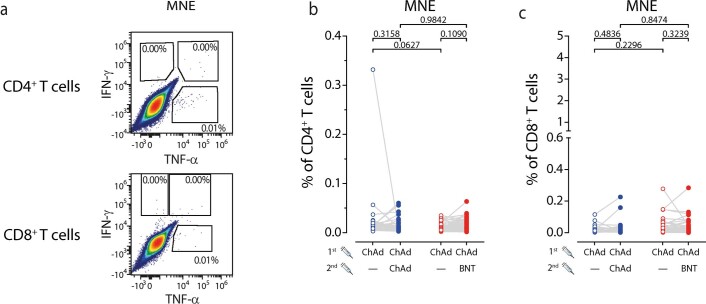
T cell cytokine production after re-stimulation with a mixture of peptides from membrane (M), nucleocapsid (N), and envelope (E) SARS-CoV-2 proteins. The total number of cytokine secreting cells was calculated as the sum of IFN-γ^+^TNF-α^-^, IFN-γ^+^TNF-α^+^, and IFN-γ^-^TNF-α^+^ cells gated as shown in (**a**) for CD4^+^ (**b**) and CD8^+^ (**c**) T cells. Data are from n = 32 biologically independent sample pairs from the ChAd/ChAd group and n = 55 biologically independent sample pairs from the ChAd/BNT group. Paired t-test (within groups) or 2-way ANOVA followed by Sidak’s multiple comparison test (between groups). Source data

In addition to humoral immune responses, we also analyzed frequencies and phenotypes of spike-specific T cells (Methods and Extended Data Fig. 7). The frequencies of spike-specific CD4^+^ T cells in blood samples collected before booster vaccination were significantly higher for both vaccination groups compared to the MNE (control) peptides or DMSO alone (Fig. 2a and Extended Data Fig. 8). No significant differences were found between the ChAd/ChAd and ChAd/BNT groups (Fig. 2a, open circles). After boosting, the frequencies for spike-specific CD4^+^ T cells increased in both groups and were significantly higher in the ChAd/BNT group (Fig. 2a, filled dots). The same effect was observed for spike-specific CD8^+^ T cells. These cells were present at similar frequencies in both groups before boosting and increased in frequencies after boosting. Again, boosting with BNT induced higher frequencies than boosting with ChAd (Fig. 2b, filled dots). Regarding the distribution of spike-specific CD8^+^ T cells producing interferon (IFN)-γ or tumor necrosis factor (TNF)-α, application of both booster vaccines led to an increase in the proportion of cells producing both cytokines simultaneously (Fig. 2c). Significant increase in spike-specific IFN-γ-producing T cells in the ChAd/BNT group but not in the ChAd/ChAd group was confirmed by cytokine measurement in supernatants after SARS-CoV-2 spike peptide stimulation (Fig. 2d).

Due to the abrupt recommendation of several European governments to discontinue the use of ChAd in the young and middle-aged population, a unique situation was created in which heterologous prime-boost vaccination regimens were applied despite the lack of any information available regarding immunogenicity and safety aspects. This study provides insights into the immunogenic outcome of homologous and heterologous vaccination protocols with two vaccines: BNT and ChAd. Head-to-head comparison of ChAd-primed vaccinees who received either a ChAd or BNT booster immunization revealed that both regimens enhanced both humoral and cellular immune responses. Although this setup did not allow for randomization of the participants, and we are, thus, unable to completely exclude confounding factors, our study revealed that the group boosted with BNT showed significantly stronger immune responses than the group boosted with ChAd. CD4^+^ and CD8^+^ T cell responses directed against spike protein epitopes were higher in frequencies, and cells produced more IFN-γ upon re-stimulation. Likewise, the group boosted with BNT developed higher titers of anti-spike protein antibodies of both the IgG and IgA subclasses, and these differences were not significantly influenced by age or sex. It should be noted that these antibodies were highly efficient in neutralizing all three VoC tested in the present study. It was previously reported that vaccinees immunized with BNT/BNT also develop neutralizing antibodies against the VoC^20^. We confirmed these findings in the present study using data from the participants of the CoCo Study cohort who were also immunized with BNT/BNT. Our data indicate that BNT/BNT-vaccinated and ChAd/BNT-vaccinated individuals develop neutralizing antibodies to similar degrees 2–3 weeks after booster vaccination. Likewise, immune responses of the ChAd/ChAd group were in the range of earlier reported results^11–13,21^. Although it would have been interesting to also characterize immune responses in a cohort of people immunized with BNT/ChAd, such individuals were not available to us. We want to emphasize that our data obtained in mostly healthy and relatively young HCPs cannot be generalized to elderly people or to specific patient groups. Another limitation of our study is that we were unable to test neutralizing activity against the Delta variant and to collect data on safety and reactogenicity after vaccination.

Extended studies, ideally including clinical endpoints, are needed to further characterize immune responses not only in heterologously immunized cohorts. It will be of particular importance to examine neutralizing activity against novel VoC, such as the Delta variant, and how long protective immune responses are maintained, in both individuals who are at elevated risk for developing severe COVID-19 and individuals who are known for mounting impaired immune responses.

## Methods

### Participants

Participants for this analysis were from the CoCo Study (German Clinical Trial Registry, DRKS00021152), which started in March 2020 and is an ongoing, prospective, observational study monitoring anti-SARS-CoV-2 IgG immunoglobulin and immune responses in 1,493 HCPs at Hannover Medical School and in individuals with potential contact to SARS-CoV-2 (refs. ^18,22^). An amendment from December 2020 allowed us to study the immune responses after COVID-19 vaccination. According to German regulations, HCPs were prioritized for SARS-CoV-2 vaccination, and HCPs at Hannover Medical School received first doses of either the BNT vaccine after 6 January or the ChAd vaccine after 16 February 2021. In general, booster vaccination took place approximately 21 d after BNT prime and 2–3 months after ChAd prime. Booster vaccination of ChAd-primed HCPs started on 3 May 2021, and individuals could choose to receive either ChAd or BNT for second vaccination. We assumed that about 25% of all ChAd-primed vaccinees would opt for a homologous booster. The power calculation, performed with G*Power (v3.1.9.6), determined that a sample size of 30 individuals in each arm is sufficient to detect clinically meaningful differences within each group, assuming that spike protein-specific IgGs double from first vaccination (mean, 95 relative units (RU) ml^−1^, with an s.d. of 113 RU ml^−1^) and when using a two-tailed paired *t*-test for differences between means with a 95% power and a 1% level of significance. Based on the above calculations and an expected loss to follow-up rate of 10%, 130 ChAd-primed vaccinees of the CoCo Study cohort were invited to donate blood before their boosting started in early May 2021. Scheduling appointments for vaccination was coordinated by an independent vaccination team according to vaccine availability. After written informed consent, we obtained peripheral blood samples by venipuncture. On a first-come, first-served basis, we performed our formal statistical analysis once at least 30 individuals in each arm had received a booster vaccination and had passed day 13 after booster. One individual with previous SARS-CoV-2 infection, as determined by positive anti-SARS-CoV-2 nucleocapsid (NCP) IgG before vaccinations, was excluded from this analysis. Participants were 25% male and 75% female, with a mean age of 38 years (range 19–64 years) and, thus, representative of all vaccinees of the CoCo Study (72% female, 28% male; mean age 40 years, range 19–67 years). After blood collection, we separated plasma from EDTA or lithium heparin blood (S-Monovette, Sarstedt) and stored it at −80 °C until use. For stimulation with SARS-CoV-2 peptide pools, we used full blood or peripheral blood mononuclear cells (PBMCs) from whole-blood samples isolated by Ficoll gradient centrifugation.

### pVNT

pVNTs were performed at the Infection Biology Unit of the German Primate Center in Göttingen as described previously^12^. Briefly, the rhabdoviral pseudotyped particles were produced in 293T cells transfected to express the desired SARS-CoV-2-S variant inoculated with VSV*DG-FLuc, a replication-deficient vesicular stomatitis virus (VSV) vector that encodes for enhanced green fluorescent protein and firefly luciferase (FLuc) instead of VSV-G protein (kindly provided by Gert Zimmer, Institute of Virology and Immunology, Mittelhäusern, Switzerland). Produced pseudoparticles were collected, cleared from cellular debris by centrifugation and stored at −80 °C until used. For neutralization experiments, equal volumes of pseudotyped particles and heat-inactivated (56 °C, 30 min) plasma samples serially diluted in culture medium were mixed and incubated for 30 min at 37 °C. Afterwards, the samples, together with non-plasma-exposed pseudotyped particles, were used for transduction experiments. The assay was performed in 96-well plates in which Vero cells were inoculated with the respective pseudotyped particles/plasma mixtures. The transduction efficacy was analyzed at 16–18 h after inoculation by measuring FLuc activity in lysed cells (Cell Culture Lysis Reagent, Promega) using a commercial substrate (Beetle-Juice, PJK) and a plate luminometer (Hidex Sense Microplate Reader, Hidex) with the Hidex Sense Microplate Reader Software (v0.5.41.0).

### Serology

We determined SARS-CoV-2 IgG serology by quantitative ELISA (anti-SARS-CoV-2 S1 spike protein domain/receptor-binding domain (RBD) IgG SARS-CoV-2 QuantiVac, Euroimmun) according to the manufacturer’s instructions (dilution 1:400 or 1:600). We provide antibody levels expressed as RU ml^−1^ as assessed from a calibration curve, with values above 11 RU ml^−1^ defined as positive. We performed anti-SARS-CoV-2 S1 spike protein domain IgA or anti SARS-CoV-2 NCP IgG measurements according to the manufacturer’s instructions (Euroimmun) and expressed antibody amounts as IgA ratio (optical density (OD) divided by calibrator). We used an AESKU.READER (AESKU.GROUP) and the Gen5 version 2.01 software for analysis.

### sVNT for SARS-CoV-2 variants

To determine neutralizing antibodies against the Wuhan spike, the B.1.1.7-spike (Alpha), the P.1-spike (B.1.1.28.1; Gamma) and the B.1.351-spike (Beta) variants of SARS-CoV-2-S in plasma, we modified our recently established sVNT^19^. In this assay, the soluble receptor for SARS-CoV-2—angiotensin-converting enzyme 2 (ACE2)—is bound to 96-well-plates to which different purified tagged RBDs of the spike protein of SARS-CoV-2 can bind once added to the assay. Binding is further revealed by an anti-tag peroxidase-labelled antibody and colorimetric quantification. Pre-incubation of the spike protein with serum or plasma of convalescent patients or vaccinees prevents subsequent binding to ACE2 to various degrees, depending on the amount of neutralizing antibodies present. In detail, Maxisorp 96F plates (Nunc) were coated with recombinant soluble hACE2-Fc(IgG1) protein at 300 ng per well in 50 μl of coating buffer (30 mM Na_2_CO_3_, 70 mM NaHCO_3_, pH 9.6) at 4 °C overnight. After blocking with hACE2-Fc(IgG1), plates were washed with PBS, 0.05% Tween-20 (PBST) and blocked with BD OptEIA Assay Diluent for 1.5 h at 37 °C. In the meantime, plasma samples were serially diluted three-fold starting at 1:20 and then pre-incubated for 1 h at 37 °C with 1.5 ng of recombinant SARS-CoV-2 spike RBD of the Wuhan strain (Trenzyme), the B.1.1.7 variant (N501Y; Alpha), the B.1.351 variant (K417N, E484K, N501Y; Beta) or the P.1 variant (K417T, E484K, N501Y; Gamma) (the latter three from SinoBiological), all with a C-terminal His-tag. BD OptEIA Assay Diluent was used for preparing plasma sample as well as RBD dilutions. After pre-incubation with SARS-CoV-2 spike RBDs, plasma samples were given onto the hACE2-coated Maxisorp ELISA plates for 1 h at 37 °C. SARS-CoV-2 spike RBDs pre-incubated with buffer only served as negative controls for inhibition. Plates were washed three times with PBST and incubated with an horseradish peroxidase (HRP)-conjugated anti-His-tag antibody (clone HIS 3D5, provided by Helmholtz Zentrum München) for 1 h at 37 °C. Unbound antibody was removed by six washes with PBST. A colorimetric signal was developed on the enzymatic reaction of HRP with the chromogenic substrate 3,3′,5,5′-tetramethylbenzidine (BD OptEIA TMB Substrate Reagent Set). An equal volume of 0.2 M H_2_SO_4_ was added to stop the reaction, and the absorbance readings at 450 nm and 570 nm were acquired using a SpectraMax iD3 microplate reader (Molecular Devices) using SoftMax Pro version 7.03 software. For each well, the percent inhibition was calculated from OD values after subtraction of background values as: Inhibition (%) = (1 − Sample OD value/Average SARS-CoV-2 S RBD OD value) × 100. Neutralizing sVNT titers were determined as the dilution with binding reduction > mean + 2 s.d. of values from a plasma pool consisting of three pre-pandemic plasma samples.

### SARS-CoV-2 protein peptide pools

We ordered 15 amino acid (aa)-long and 10 aa-overlapping peptide pools spanning the whole length of SARS-CoV2-Spike (-S) (total 253 peptides), -Membrane (-M) (total 43 peptides), -Nucleocapsid (-N) (total 82 peptides) or -Envelope (-E) (total 12 peptides; peptide no. 4 could not be synthesized) from GenScript. All lyophilized peptides were synthesized at greater than 95% purity and reconstituted at a stock concentration of 50 mg ml^−1^ in DMSO (Sigma-Aldrich), except for nine SARS-CoV2-S overlapping peptides (nos. 24, 190, 191, 225, 226, 234, 244, 245 and 246), two for SARS-CoV2-M (nos. 15 and 16), one for SARS-CoV2-N (no. 61) and all 12 SARS-CoV2-E peptides that were dissolved at 25 mg ml^−1^ due to solubility issues. All peptides in DMSO stocks were stored at −80 °C until used.

### T cell re-stimulation assay

PBMCs, isolated using a Ficoll gradient, were resuspended at a concentration of 20 × 10^6^ cells per ml in complete RPMI medium (RPMI 1640 (Gibco)) supplemented with 10% FBS (GE Healthcare Life Sciences), 1 mM sodium pyruvate, 50 µM β-mercaptoethanol and 1% streptomycin–penicillin (all Gibco). For stimulation, cells were diluted with equal volumes of peptide pools containing S-protein or mixture of M-, N- and E-proteins. Peptide pools were prepared in complete RPMI containing brefeldin A (Sigma-Aldrich) at a final concentration of 10 µg ml^−1^. In the final mixture, each peptide had a concentration of 2 µg (~1.2 nmol) ml^−1^, except for SARS-CoV2-S peptides 24, 190, 191, 225, 226, 234, 244, 245 and 246, SARS-CoV2-M peptides 15 and 16 and SARS-CoV2-N peptide 61, which were used at a final concentration of 1 µg ml^−1^ due to solubility issues. As a negative control, we stimulated the cells with DMSO, used in maximal volume corresponding to DMSO amount in peptide pools (equaling to 5% DMSO in final medium volume). In each experiment, we used cells stimulated with phorbol-12-myristate-13-acetate (Calbiochem) and ionomycin (Invitrogen) at a final concentration of 50 ng ml^−1^ and 1,500 ng ml^−1^, respectively, as an internal positive control. Cells were then incubated for 12–16 h at 37 °C, 5% CO_2_. After washing, cells were resuspended in MACS buffer (PBS supplemented with 3% FBS and 2 mM EDTA). Non-specific antibody binding was blocked by incubating samples with 10% mouse serum at 4 °C for 15 min. Next, without washing, an antibody mix of anti-CD3-AF532 (UCHT1; no. 58-0038-42, lot no. 2288218; Invitrogen; 1:50), anti-CD4-BUV563 (RPA-T4; no. 741353, lot no. 9333607; BD Biosciences; 1:200), anti-CD8-SparkBlue 550 (SK1; no. 344760, lot no. B326454; BioLegend; 1:200), anti-CD45RA (HI100; no. 740298, lot no. 0295003; BD Biosciences; 1:200), anti-CCR7 (G043H7; no. 353230, lot no. B335328; BioLegend; 1:50), anti-CD38 PerCP-eF710 (HB7; no. 46-0388-42, lot no. 2044748; Invitrogen; 1:100) and Zombie NIR Fixable Viability Kit (no. 423106, lot no. B323372; BioLegend) was added. After staining for 20 min at room temperature, cells were washed before they were fixed and permeabilized (no. 554714, BD Biosciences) according to the manufacturer’s protocol. Next, intracellular cytokines were stained using anti-IFN-PE-Cy7 (B27; no. 506518, lot no. B326674; BioLegend; 1:100), anti-TNF-AF700 (Mab11; no. 502928, lot no. B326186; BioLegend; 1:50) and anti-IL-17A-BV421 (BL168; no. 512322, lot no. B317903; BioLegend; 1:50) for 45 min at room temperature. Excess antibodies were washed away, and cells were then acquired on a Cytek Aurora spectral flow cytometer (Cytek) equipped with five lasers operating at 355 nm, 405 nm, 488 nm, 561 nm and 640 nm. All flow cytometry data were acquired using SpectroFlo version 2.2.0 (Cytek) and analyzed with FCS Express 7 (Denovo).

### Flow cytometric analysis of spike-specific B cells

Total leukocytes were isolated from whole blood using erythrolysis in 0.83% ammonium chloride solution. Isolated cells were then washed, counted and resuspended in PBS and stained for 20 min at room temperature with an antibody mix containing antibodies listed in Extended Data Fig. 4 together with spike-mNEONGreen protein (5 μg per reaction; production will be described elsewhere). After one wash, samples were acquired on a spectral flow cytometer, and the data were analyzed as described above.

### Quantification of IFN-γ and TNF-α release

Next, 0.5 ml of full blood was stimulated with manufacturer’s selected parts of the SARS-CoV-2 S1 domain of the spike protein for a period of 20–24 h. We carried out negative and positive controls according to the manufacturer’s instructions (SARS-CoV-2 Interferon Gamma Release Assay (Euroimmun)). After stimulation, supernatants were collected after centrifugation and examined by the LEGENDplex kit (BioLegend) according to the manufacturer’s instructions. Data from duplicate measurements were acquired with a LSR II flow cytometer (BD Biosciences) using BD’s FACSDiva version 8.0.1 software and analyzed with the LEGENDplex Data Analysis Software Suite, Gen5 version 2.01 software.

### Statistics

Statistical analysis was performed using GraphPad Prism version 8.4 (GraphPad Software) and SPSS version 20.0.0 (IBM, SPSS Statistics). For comparison of spike-specific IgG and IgA levels, as well as for comparison of percentages of cytokine-secreting T cells or cytokine concentrations in serum, we used the paired *t*-test (within groups) or two-way analysis of variance (ANOVA) followed by Sidak’s multiple comparison test (between groups). For comparison of sVNT titers, we used the chi-square test for trend. Differences were considered significant if *P* < 0.05. Correlation between sVNT and pVNT values was calculated using single linear regression analysis.

### Ethics committee approval

The CoCo Study and the analysis conducted for this article were approved by the Internal Review Board of Hannover Medical School (institutional review board no. 8973_BO-K_2020, amendment December 2020).

### Reporting Summary

Further information on research design is available in the Nature Research Reporting Summary linked to this article.

## Online content

Any methods, additional references, Nature Research reporting summaries, source data, extended data, supplementary information, acknowledgements, peer review information; details of author contributions and competing interests; and statements of data and code availability are available at 10.1038/s41591-021-01449-9.

## Supplementary information

Reporting Summary

## Data Availability

All requests for raw and analyzed data that underlie the results reported in this article will be reviewed by the CoCo Study team at Hannover Medical School (cocostudie@mh-hannover.de) to determine whether the request is subject to confidentiality and data protection obligations. Data that can be shared will be released via a material transfer agreement. Source data are provided with this paper.

## Source data

Source Data Fig. 1 Statistical source data.
Source Data Fig. 2 Statistical source data.
Source Data Extended Data Fig. 2 Statistical source data.
Source Data Extended Data Fig. 3 Statistical source data.
Source Data Extended Data Fig. 5 Statistical source data.
Source Data Extended Data Fig. 6 Statistical source data.
Source Data Extended Data Fig. 8 Statistical source data.
